# The player salary costs of match-loss injury and illness at an Australian Football League Club: A six-season retrospective cohort study

**DOI:** 10.1016/j.jsampl.2025.100104

**Published:** 2025-06-04

**Authors:** Matthew R. Turnbull, Tania F. Gallo, Hannah E. Carter, Michael Drew, Liam A. Toohey, Jocelyn Mara, Gordon Waddington

**Affiliations:** 1 University of Canberra Research Institute for Sport and Exercise (UCRISE), Canberra, Australia; 2 Cricket Australia, Melbourne, Australia; 3 Australian Centre for Health Services Innovation and Centre for Healthcare Transformation, School of Public Health and Social Work, Faculty of Health, Queensland University of Technology, Brisbane, Australia; 4 Australian Institute of Sport, Bruce, Australia

**Keywords:** Athletic injuries, Financial cost, Health economics, Prevention

## Abstract

**Background:**

The objective of this study was to quantify the salary costs of match-loss injuries and illnesses at a single professional AFL club.

**Methods:**

A retrospective cohort study involving male professional AFL players across a six-season period (2016–2021). Analysis of player injury and illness data, and corresponding salary data, was performed with costs calculated using the human capital method.

**Results:**

There were 95 individual players across the six-seasons, with 79 unique diagnoses, 267 match-loss injuries or illnesses and 1130 matches missed. The total salary cost of match-loss injury and illness was AU$13.0 million across the period. Hamstring biceps femoris grade 1–2 strains had the highest proportion of these costs according to diagnosis (AU$1.3 million, 30 incidences, 10.0 ​% of total salary costs), followed by soleus strains (AU$1.1 million, 20 incidences, 8.7 ​%) and concussions (AU$1.1 million, 25 incidences, 8.4 ​%). Of the most frequent injuries, hamstring semimembranosus strains had the highest mean (SD) cost per injury occurrence at AU$139,988 (126,023), followed by knee anterior cruciate ligament injuries at AU$99,264 (105,086). Injury or illness costs as a proportion of the total salary spend across six seasons was 17 ​% (range 10 ​%–22 ​%).

**Conclusion:**

Match-loss injuries and illnesses incur a considerable financial cost relative to the player salary expenditure. This study may provide a costing model for sports businesses to utilise as an approach to inform resource allocation decisions, particularly in relation to injury management and prevention.

## 1 Background

Australian Rules football has been reported as the most popular football code in Australia [1,2]. The national professional competition, the Australian Football League (AFL) is associated with high injury rates [3]. In 2023, an injury incidence of 34.9 new time-loss injuries per club per season was reported, with 135.7 matches missed per club per season [4]. Athletes being unavailable to participate in sport due to injury or illness is associated with a range of financial costs including direct healthcare-related expenses [5] and indirect lost productivity costs due to time-loss from the sport [[6], [7], [8], [9], [10], [11]]. Quantifying those costs may provide valuable information to inform decision-making practices across the various sociological levels within the AFL and other professional sports settings.

The financial costs of injuries and illnesses in the AFL are largely unknown. The salary costs of missing an AFL match due to injury has been estimated in one study [10], which specifically focused on hamstring injuries. This study identified hamstring injury data from the AFL injury report [12,13] and then proportioned a weekly salary cost per injury from data obtained from AFL annual reports [14] to estimate the annual salary cost per hamstring injury over a 10 year period (AU$25,603 in 2003 to AU$40,021 in 2012). The salary costs of match-loss injury have been estimated in a number of other professional sport study settings including Major League Baseball (MLB) [6], the National Hockey League (NHL) [7,9], the National Basketball Association (NBA) [11] and the National Football League (NFL) [8]. The aforementioned studies used similar methods, estimating the salary costs of match-loss injury using top-down approaches to proportion costs from known or estimated total salary expenditures derived from publicly available resources [15]. The limitation of these studies is that they potentially overestimate or underestimate the true athlete salary costs of a match-loss injury as individual athletes may be on disparate incomes, or on different contract agreements. The actual player payment costs of missing an AFL match due to injury or illness have not been reported.

The AFL enforces a salary cap to limit the amount each club is able to spend on player salaries, as a means to improve competitive balance within the competition [1]. We hypothesise that the constraints on an AFL club's player salary spend may be considerably compounded by the indirect salary costs of injury or illness and that these costs may contribute a significant proportion of the annual AFL salary cap. Understanding the extent to which specific match-loss injuries or illnesses impact financially on a professional club may benefit decision makers within AFL clubs, the AFL and wider professional sports businesses. These benefits may include the prioritisation of prevention planning, the distribution of healthcare resources, and to inform policy initiatives. At an AFL competition level, it may also influence funding policy related to the healthcare of athletes.

The purpose of this study was to quantify the salary costs of match-loss injuries and illnesses at a single club within the AFL competition.

## 2 Materials and methods

### 2.1 Study design, cost perspective and type of costs

This was a retrospective cohort study of male professional AFL players contracted to an AFL club during a six-year period (2016–2021). The study explored the cost perspective of a single AFL club and only salary costs associated with match-loss injury or illness were calculated. This study did not quantify the costs associated with replacing an injured player and did not include direct medical costs associated with the prevention or treatment of injuries or illnesses.

### 2.2 Ethics

Ethics approval was granted by the University of Canberra Human Research and Ethics Committee (approval number 3337). Consent was granted by the club for player data from 2016 to 2021 to be used for research and publication purposes. In addition to the anonymisation of data, to enhance protection of player privacy the researchers signed non-disclosure agreements compiled by the club and ensured only aggregated results where players could not be identified were reported.

### 2.3 Data collection, injury, and illness definition

Systematically collected injury and illness data were prospectively recorded by club medical staff and compiled in a ‘Player Movement Report’. Injuries were recorded using the ‘match-loss injury and illness’ definition [16] (Table 1) and assigned an Orchard Sports Injury Classification Score (OSICS) 10.1 diagnostic code [17]. Body area and injured structure were included from the Player Movement Report. Financial data were documented in the ‘Player Payment Record’. Player payment mechanisms were determined from the Player Payment Record and presented narratively to explain how players were remunerated. De-identified extracts from both reports were provided to the research team. To assist the interpretation of the methods, key definitions are listed in Table 1.

**Table 1:** Key definitions and data items.

Match-loss injury or illness	Any “injury or medical condition which causes a player to miss a match” [16]. This included any injuries or illnesses to a contracted player that resulted in a missed match, regardless of whether the injury or illness occurred at training, during a match or elsewhere. An injury or illness was considered to be resolved when a player returned to play regardless of AFL match or state league match selection.
Indirect costs	The costs incurred due to a loss of productive output due to disability or premature death [18]. In the context of this study, these productivity costs are referred to as salary costs attributable to injury or illness.
Human capital method	Considers employees hours of productivity that are lost due to absenteeism and designed to estimate the value of human capital by calculating productivity costs as a product of those total lost hours with the hourly wage [18]. In the context of this study, absenteeism is a missed match due to injury or illness.
Player movement report	An electronic injury and illness record that is submitted by the club to the AFL each season for the annual AFL injury ​report. [13,19] included in this report are the season, player, the match-loss injury or illness diagnosis, body area, injured structure, injury date, return to play date and number of matches missed.
Player payment record	An electronic record of individual player salaries per season containing the breakdown of player payments including base salaries, total match payments and additional payments.
Player base salaries	The annual salary paid to an individual player before match payments and additional payments.
Total match payments	The sum of match related payments that are paid to players on top of their base salaries including: Match payments, injury payments, AFL emergency payments, rested payments and medical-sub payments received per player.
Additional payments	Additional payments made to players including benefits, incentives, additional service agreements and ‘other’ payments.

### 2.4 Data analysis

#### 2.4.1 Cost of match-loss injury and illness diagnoses

Base salaries and total match payments were included in the cost per match-loss injury and illness diagnosis analysis. Additional payments were excluded as it was not possible to consistently link additional payment items such as benefits, performance incentives, additional service agreements to match-loss injury or illness. The human capital method (Table 1) was used, based on the one match per week AFL fixture, to calculate the costs of match-loss injury and illness. This involved (1) calculating the sum of individual base salaries and the total match payments received per player, per season; (2) then establishing a weekly salary per player by dividing the sum of the base salaries and the total match payments by the number of matches the team played per season, (3) and then calculating the cost of match-loss injury or illness by multiplying the weekly player salary by the number of matches missed per player per season. R version 4.4.2 in RStudio version 2024.04.2 ​+ ​764 was used for all analysis. Results of this calculation are reported as descriptive statistics: cumulative sums, mean, standard deviations (SD), median, first and third quartiles.

#### 2.4.2 The impact of match-loss injury and illness cost

To establish the impact of injury and illness costs, descriptive statistics were presented as a percentage of the club's total player salary spend each season, including additional payments. The inclusion of additional payments to this part of the analysis was required in order to capture the total expenditure and not just the injury related costs. Under AFL rules [20], clubs cannot exceed the total player payment (TPP) limit. However, certain deductions including ‘benefits’ payments such as player relocation expenses and certain ‘rookie player’ salaries are permitted. Deductions were excluded from the analysis to capture total salary expenditure and therefore results illustrated in this study exceed the TPP limit.

#### 2.4.3 The 2020 Covid-19 season

In 2020, player salaries were reduced by 50 ​% for the period following round one as a result of the Covid-19 pandemic. That is, all match-loss related payments including base salaries and match payments were paid at 100 ​% up to and including round one, however from round 2–18 of the Covid-19 modified 2020 AFL season, player payments were paid at 50 ​% of the original amount. The 2020 season also had less regular season matches played per season (17 matches) compared to the other seasons (22 matches). This season represents an outlier that could bias the results to report lower than expected totals and should be considered by the reader when interpreting reported averages in the results. For instance, when observing the 2020 season injury and illness costs as a proportion of the total expenditure, the results may be considered 23 ​% lower, explained by the five less matches (23 ​% less matches played) that season.

## 3 Results

### 3.1 Population and injury or illness characteristics

Ninety-five professional male players were contracted to the club over six consecutive seasons (2016–2021). Mean (SD) player age was 23.8 (4.2), weight (kg) 87.3 (8.4) and height (cm) 189.0 (6.5). There were 44.8 (0.8) players at the club per season, of which 28 (3.0) players sustained at least one match-loss injury or illness that resulted in 6.7 matches missed per injured player each season (Supplement 1). Seventy-nine unique injury or illness diagnoses were recorded, totalling 267 match-loss injuries or illnesses that resulted in 1130 matches missed over the six seasons.

### 3.2 Player payment mechanisms: player payments disparity

Player contractual arrangements varied across the cohort, impacting injury and illness remuneration. For instance, a hamstring strain injury in a higher earning player was more expensive when compared to the same injury in the lower earning player. Furthermore, all players received a base salary, however not all players received match payments or additional payments, and only those on match payment agreements received injury payments for missed matches due to injury or illness. When a player on a match-payment agreement was injured during a match, he continued to receive an ‘injury payment’ for every subsequent missed match at the ‘match payment’ rate until his return to competition. Across the six seasons the majority (95 ​%, n ​= ​58) of players who received injury payments were due to match-loss injuries that occurred during AFL matches. In some circumstances players received injury payments for injuries sustained during a state league match, however this only occurred with three players across the six seasons (5 ​%, n ​= ​3).

### 3.3 Cost of match-loss injury and illness

The total salary cost of match-loss injury and illness was AU$13.0 million over the six seasons, with an average annual cost of AU$2.2 million (SD ​= ​553,295). Joint injuries made up the greatest proportion of the total injury and illness costs per body structure, followed by muscle and bone injuries (Table 2). The knee joint was the costliest body area followed by hamstring injuries (Table 3). Notably, medical illness had a relatively low total cost AU$225,826, with only 10 illnesses that resulted in 16 missed matches over the six seasons (Table 3).

**Table 2:** Total cost per body structure injured.

Injured structure	Matches missed	Total cost (AUD)	Mean (SD) cost per area (AUD)	Median cost per area (AUD)	25th percentile (AUD)	75th percentile (AUD)
**Joint**	455	$4,981,173	$55,346 (73,703)	$25,147	$11,443	$64,168
**Muscle**	299	$4,015,502	$45,631 (54,840)	$21,985	$13,240	$53,693
**Bone**	229	$2,150,866	$58,132 (52,727)	$40,513	$23,864	$68,182
**Nervous system**	75	$1,097,413	$43,897 (92,137)	$15,352	$9545	$26,364
**Tendon**	55	$530,627	$33,164 (40,957)	$16,924	$6465	$34,773
**Other**	17	$242,372	$22,034 (26,370)	$14,860	$5354	$24,775

AUD ​= ​Australian dollars; SD ​= ​standard deviation.

**Table 3:** Total costs per injured body area or medical illness.

Injury	Number	Matches missed	Mean matches missed per injured area	Total cost (AUD)	Mean (SD) cost per area (AUD)	Median cost per area (AUD)	25th percentile (AUD)	75th percentile (AUD)
Knee	36	206	5.7	$2,132,531	$59,237 (75,720)	$19,000	$8567	$87,405
Hamstring	40	160	4.0	$2,043,473	$51,087 (60,281)	$29,591	$15,375	$56,203
Foot	24	133	5.5	$1,713,400	$71,392 (88,849)	$41,734	$18,432	$90,182
Ankle	20	136	6.8	$1,326,414	$66,321 (45,202)	$47,198	$37,469	$92,672
Calf	22	78	3.5	$1,151,700	$52,350 (60,841)	$21,985	$17,166	$69,545
Head	25	75	3.0	$1,097,413	$43,897 (92,137)	$15,352	$9545	$26,364
Shoulder	22	63	2.9	$652,418	$29,655 (39,014)	$17,409	$7430	$33,927
Lumbar spine	11	64	5.8	$651,024	$59,184 (87,230)	$18,698	$11,136	$65,909
Other	11	56	5.1	$463,385	$42,126 (56,093)	$23,864	$9409	$47,557
Pelvis	7	44	6.3	$422,924	$60,418 (66,921)	$33,250	$16,000	$70,905
Adductor	9	22	2.4	$355,431	$39,492 (33,686)	$20,652	$15,352	$53,864
Hand	8	28	3.5	$355,407	$44,426 (36,175)	$27,864	$19,773	$74,318
Quadriceps	12	27	2.2	$314,910	$26,242 (15,911)	$24,020	$18,772	$30,883
Medical illness	10	16	1.6	$225,826	$22,583 (27,731)	$12,203	$4745	$26,478
Hip	10	22	2.2	$111,697	$11,170 (8521)	$7273	$7120	$9699

Note: Total costs, mean and median values include the 2020 outlier season that may bias the results to a lower amount and should be considered when interpreting these results.

AUD ​= ​Australian dollars; SD ​= ​standard deviation.

The injuries or illnesses associated with the highest total cost according to diagnosis were hamstring biceps femoris grade 1–2 strains at AU$1.3 million (n ​= ​30, matches missed ​= ​117), followed by soleus strains at AU$1.1 million (n ​= ​20, matches missed ​= ​75) and concussions at AU$1.1 million (n ​= ​25, matches missed ​= ​75) (Table 4).

**Table 4:** Top eight most costly match-loss injury and illness diagnoses.

Injury or illness diagnosis	Number	Matches missed	Mean matches missed per injury	Total cost (AUD)	Mean (SD) cost per injury occurrence (AUD)	Median cost per injury (AUD)	25th percentile (AUD)	75th percentile (AUD)
Hamstring biceps femoris grade 1–2 strain	30	117	3.9	$1,314,877	$43,829 (42,313)	$29,591	$16,136	$54,318
Soleus injury strain	20	75	3.8	$1,126,549	$56,327 (62,487)	$23,348	$17,722	$85,284
Concussion	25	75	3.0	$1,097,413	$43,897 (62,487)	$15,352	$9545	$26,364
Knee acute ACL injury	8	95	11.9	$794,116	$99,264 (105,086)	$59,416	$16,973	$162,080
Ankle syndesmosis sprain	10	70	7.0	$756,011	$75,601 (55,358)	$51,198	$39,446	$103,774
Hamstring semimembranosus strain	4	30	7.5	$559,951	$139,988 (126,023)	$134,719	$33,994	$240,713
Lumbar disc injury	7	37	5.3	$541,393	$77,342 (104,639)	$39,559	$16,167	$81,864
Knee acute PCL injury	5	42	8.4	$451,423	$90,285 (57,050)	$83,767	$51,176	$111,000

Note: Total costs, mean and median values include the 2020 outlier season that may bias the results to a lower amount and should be considered when interpreting these results.

Note: Only match-loss injury and illness diagnoses with four or more incidences were included a means to protect player identity.

AUD ​= ​Australian dollars; SD ​= ​standard deviation; ACL ​= ​anterior cruciate ligament, PCL ​= ​posterior cruciate ligament.

While more frequent injury types had a higher total cost, other injuries were more costly per injury occurrence (Table 4). Of the most frequent injuries, hamstring semimembranosus strains had the highest mean (SD) cost per injury occurrence at AU$139,988 (126,023), followed by knee acute ACL injuries at $99,264 (105,086) (Table 4). When examining injuries by body area, foot injuries had the highest overall cost per occurrence at a mean (SD) of $71,392 (88,849), followed by ankle injuries at a mean of $66,321 (45,202) (Table 3).

### 3.4 The financial impact of match-loss injury and illness

The cost of match-loss injuries and illnesses as a proportion of the total player salary spend across six seasons was 17 ​% (Table 5). Season 2018 had the lowest cost and percentage (10 ​%) against the total annual salary spend, while also having the lowest number of matches missed (Table 5). The relative proportion of match-loss injury and illness costs was greatest in 2020 (22 ​%). When interpreting this proportion, consideration should be placed on the 2020 season which was an outlier due to the condensed fixture (18 rounds, 17 matches played) and players earning 50 ​% less income for the majority of the season (16 of the 17 matches played) which may inflate the proportion of injury and illness cost. However, the 2020 season had the second highest matches missed (23 ​%) proportionate to the total matches each season (Table 5).

**Table 5:** Injury and illness costs as a proportion of the annual salary spend.

Season	Rounds played	Injury Incidences	Missed matches	Total matches (% missed)^c^	Total player salaries excluding TPP deductions (AUD)	Match-loss injury and illness cost (AUD)	Proportion of total player salaries (%)
2016	23^a^	47	165	1012 (16 ​%)	$10,834,562	$1,799,560	17 ​%
2017	22	55	207	990 (21 ​%)	$13,487,205	$2,133,426	16 ​%
2018	22	43	147	990 (15 ​%)	$13,476,743	$1,302,556	10 ​%
2019	22	45	238	1001 (24 ​%)	$15,397,698	$2,756,575	18 ​%
2020	17^b^	31	173	765 (23 ​%)	$10,607,270	$2,339,140	22 ​%
2021	22	46	200	942 (21 ​%)	$13,931,870	$2,686,697	19 ​%
**Total**	**128**	**267**	**1130**	**5700 (20 ​%)**	**$77,735,348**	**$13,017,954**	**17 ​%**

AUD ​= ​Australian dollars; TPP ​= ​total player payments.

a 2016 season included one finals match and therefore included an additional round.

b 2020 season was a condensed fixture due to the Covid-19 pandemic.

c Total matches in 2019 and 2021 were adjusted to include players who entered the club in the mid-season draft available to part of the season.

## 4 Discussion

There are substantial costs associated with match-loss injuries and illnesses in professional AFL, accounting for 10–22 ​% of the annual player salary spend, over a six-year period.

Of the 79 unique diagnoses in this study, hamstring biceps femoris grade 1–2 strains, soleus strains and concussions accounted for the highest proportion of total costs, while hamstring semimembranosus strain and knee acute ACL injuries had the highest costs per occurrence. Understanding injury and illness costs may benefit decision makers within AFL clubs to target healthcare management and prevention strategies in future seasons.

### 4.1 The costliest health conditions

The costliest match-loss injuries identified in this study are perhaps not surprising, as these diagnoses were also reported to have the highest incidence rates and prevalence across the AFL competition during the observation period [19,21]. For instance, the three most burdensome injuries in the AFL competition in 2021 were hamstring strains, soleus strains and concussions [21] and this study provided a monetary cost to this burden. Biceps femoris grade 1–2 strains had the highest total cost per individual diagnosis at AU$1.3 million. When analysed by body area and structure, the total of all hamstring related injuries including biceps femoris strains, semimembranosus strains, semitendinosus trains and hamstring muscle overload, amounted to a greater total cost of AU$2.0 million. This is considerable as the total cost of hamstring injuries represented half of all muscle injury costs (muscle injury costs ​= ​AU$4.0 million). Notably, whilst hamstring semimembranosus strains had a lower total cost per diagnosis when compared to biceps femoris strains grade 1–2 strains, this injury diagnosis was considerably more expensive per injury occurrence due to the high number of matches missed for each injury occurrence.

Concussions were the third most costly injury diagnosis in the study (AU$1.1 million). Unlike hamstring injuries that had a relatively consistent incidence rate in the AFL over several years [22], the incidence rate of concussions causing missed matches increased in the AFL competition during the observation period [21]. For example, the 2021 AFL injury report highlighted an increase in 2021 to 3.86 incidences per club per season, compared to 2019 (1.92) and 2020 (1.30) [21]. This may be explained by the evolution in the AFL concussion management policy over the course of the observation period in our study [21,23]. The AFL concussion guidelines evolved from a six day recovery protocol to a mandatory 12 day rehabilitation period in 2021 [24] and consequently the number of missed matches due to concussions increased [21]. Although the indirect salary costs increased, the increase return to play time potentially enhances player welfare. The AFL's investment to protect player welfare and improve long term player outcomes was made evident by the concussion policy developments and also appointing expert personnel for concussion governance [25]. A favourable economic approach to sports injury policy suggests the prolonged return to play reduces risk and potentially promotes long term cost benefit [26]. However evidence of efficacy is required to determine long term cost benefit of the AFL concussion policy and it is uncertain to what extent the AFL utilises economic evaluation to inform such policy.

### 4.2 Implications for decision making

Cost of sports injury studies and evidence for the use of economic evaluation to inform decisions such as the resource allocation of healthcare and policy in sport is limited [15]. It is also unknown to what extent AFL clubs or other professional sports utilise health economic evaluation to inform decision making practices. Implications can be drawn from the results of this study including the investment into targeted preventive and enhanced injury management strategies at a service provider level and sports organisation governance level.

Decision makers within clubs may direct healthcare resources and prevention strategies that target the costliest injuries based on the total cost per diagnosis and the cost per injury occurrence. For example, the high total cost of hamstring biceps femoris strains warrants the use of evidence-based hamstring injury prevention methods such as the exercise prescription of eccentric strengthening [27,28] or high-speed running conditioning [29] and the investment of technologies including fixed dynamometry [30,31] and global positioning system (GPS) monitoring [32] to guide and measure the effect of these methods. These interventions require resourcing (staff and material costs), time to implement [33] and the result of this analysis allows decision makers to weigh up investing in management and prevention methods.

At a sports organisation governance level, the impact of injury and illness costs may assist to inform policy. For example, changes to the AFL soft cap. The ‘soft cap’ was introduced to the AFL competition in 2015 to promote competitive balance by limiting football department spends [20,34]. While the player salary cap is separate to the football department soft cap, the direct medical costs associated with treating injured players is a cost to the football department. High performance staff within the football department are employed to manage and prevent injuries. Due to Covid-19, 2020 saw a reduction to the soft cap limit from AU$9.7 million to AU$6.2 million [35]. Despite a return to pre Covid-19 fixturing, the soft cap has not returned to pre-Covid amounts. In 2025 the soft cap limit will be AU$7,675,000, which is a AU$400,000 increase from 2024 and will then increase by AU$250,000 in 2026 and again in 2027 [36]. The 2020 and 2021 seasons saw the greatest impact of injury and illness costs as a proportion of the club's annual player expenditure. It can be hypothesised that this limit on club staff spending may place a considerable constraint on clubs' high-performance staff and medical team who are involved in the treatment of injured players. The AFL has since made concessions to the soft cap by allowing deductions to senior coach salaries and for health and well-being [35]. It is unknown to what extent the AFL utilises health economic evaluation and modelling to inform AFL soft cap policy changes. Future evidence of the direct medical expenses due to injury and illness within AFL football departments may assist the clubs and the AFL in targeting these policy changes.

## 5 Limitations

Our study focussed solely on the salary costs of lost productivity due to match-loss injury and illness, and did not include other related costs such as direct medical expenses. While only observing the salary costs is a limitation, the current study is the first observation of actual player payments costs of injury and illness in professional sport and offers a novel AFL club perspective. The human capital method accounted for missed matches only and therefore may not have captured the overall costs of injuries such as off-season injuries, training days missed or other medical attention injuries. Future research that captures both direct medical and indirect costs across the off-season, pre-season and in-season may provide a more accurate representation of the overall costs of injury or illness within the club. Furthermore, 2020 was an outlier season and the inclusion of this season may bias the results to report generally lower values. Options to inflate the 2020 expenditures to pre-covid amounts and fixturing to or leaving this season out of the analysis completely were considered. However, both options had concerns of validity and the season was included. This study is from the cost perspective of a single club in the AFL and while results may be generalisable based on correlations to AFL epidemiological data^19 21^, there may be considerable variations in incidence rates between clubs, player payment mechanisms and the disparity of player earnings. Finally, the study did not include the clubs AFL women's team (ALFW) as data was not available due to the recency of introduction of the AFLW program to the club at the time of the study. Future studies are recommended to include both the AFL and AFLW competition players.

## 6 Conclusion

This study provides new evidence on the salary costs of match-loss injury or illness at a single professional AFL club. Hamstring biceps femoris strains, soleus strains and concussions were associated with the highest cost, and the relative impact of injury and illness costs as a proportion of the club's annual salary spend was high. The costliest diagnoses are generalisable to the AFL competition as the same injuries correlated with the most burdensome in the AFL competition during the observation period. This study may provide a costing model for sports businesses to utilise as an approach to assist injury management and prevention decision making.

**Key points**

• This study provides a novel observation of the actual salary costs of match-loss injury or illness at a single professional AFL club
• The study highlights the most-costly diagnoses and the relative impact of injury and illness costs as a proportion of the club's annual salary spend
• The results are generalisable to the AFL competition as the costliest diagnosis in this study were also the most burdensome in the AFL competition during the observation period
• This study provides a costing model for sports businesses to utilise as an approach to target the costliest injuries and weigh up the investment of healthcare management and prevention strategies

## Funding

The research did not receive any specific grant from funding agencies in the public, commercial, or not-for-profit sectors.

## Declaration of competing interest

None.
