# Supplementary material for: The player salary costs of match-loss injury and illness at an Australian Football League Club: A six-season retrospective cohort study
Source: JSAMS Plus. 2025 Jun 4;5:100104. doi: 10.1016/j.jsampl.2025.100104 (PMC13008421; doi:10.1016/j.jsampl.2025.100104)
Supplement: Multimedia component 1 [file mmc1.docx]

Supplemental file 1 – Additional tables for the breakdown of population characteristics, Injury and illness cost

Table: Number of players who sustained match-loss injuries or illness per season

| **Season** | **Number of players** | **Total injury and illness incidences** | **Total matches missed** | **Number of players injured or ill (%)** | **Mean matches missed per injured or ill player (SD)** |
| --- | --- | --- | --- | --- | --- |
| 2016 | 44 | 47 | 165 | 26 (59.1%) | 6.35 (5.15) |
| 2017 | 45 | 55 | 207 | 31 (68.9%) | 6.68 (5.69) |
| 2018 | 45 | 43 | 147 | 29 (64.4%) | 5.07 (5.05) |
| 2019 | 46 | 45 | 238 | 30 (65.1%) | 7.93 (6.76) |
| 2020 | 45 | 31 | 173 | 23 (51.1%) | 7.52 (4.57) |
| 2021 | 44 | 46 | 200 | 29 (65.9%) | 6.90 (5.42) |
